# Coping Styles and General Self-Efficacy Among Pregnant Women: Evidence from a Multicenter Study in Tunisia

**DOI:** 10.3390/healthcare14131977

**Published:** 2026-07-02

**Authors:** Maha Dardouri, Nader Alnomasy, Bushra Alshammari, Shaima Mohammed Nageeb, Sonia Rouatbi, Radhia Chaieb, Imen Ayouni

**Affiliations:** 1 Community Health Nursing Department, College of Nursing, University of Hail, Hail 21424, Saudi Arabia; 2 Medical Surgical Nursing Department, College of Nursing, University of Hail, Hail 21424, Saudi Arabia; n.alnomasy@uoh.edu.sa (N.A.); bu.alshammari@uoh.edu.sa (B.A.); 3 Mental Health Nursing Department, College of Nursing, University of Hail, Hail 21424, Saudi Arabia; sm.nageeb@uoh.edu.sa; 4 Department of Physiology, University Hospital Farhat Hached, Sousse 4000, Tunisia; sonia.rouatbi@rns.tn; 5 Higher Institute of Nursing Sciences, University of Sousse, Sousse 4002, Tunisia; radhiasousse@gmail.com; 6 Department of Epidemiology, University Hospital Farhat Hached, Sousse 4000, Tunisia; eman.layouni@gmail.com

**Keywords:** stress, coping styles, self-efficacy, maternity, well-being, pregnancy

## Abstract

**Highlights:**

**What are the main findings?**

- Emotion-focused coping was the most used strategy among pregnant women, followed by problem-focused and avoidant coping.
- Higher general self-efficacy significantly predicted greater use of adaptive coping styles, including problem-focused and emotion-focused strategies.
- Social determinants, such as unemployment, divorce, rural residence, and education level, were strongly associated with variations in coping behaviors.

**What are the implications of the main findings?**

- The observed associations suggest that psychosocial screening during antenatal care may help identify women who are potentially vulnerable to psychosocial challenges, particularly those reporting limited family support.
- Counseling interventions and peer support initiatives may be considered as potential approaches to support emotional resilience and perceived social support among pregnant women, particularly those facing contextual vulnerabilities.

**Abstract:**

**Background**: Coping styles and general self-efficacy (GSE) are central to managing pregnancy challenges, yet evidence from lower-middle-income countries remains limited. This study aimed to identify coping styles during pregnancy and to assess their associations with GSE, social determinants, and pregnancy-related factors. **Methods**: A multicenter cross-sectional study was conducted among pregnant women aged 18–45 years attending antenatal clinics in Sousse, Tunisia, between July 2024 and March 2025. A multistage random sampling strategy recruited participants. Data was collected using validated Arabic versions of the Brief COPE Inventory and the General Self-Efficacy Scale (GSES). **Results**: Among 417 respondents, emotion-focused coping was the most frequently used coping strategy (mean = 31.83 ± 6.85). Mean GSE was 28.15 ± 6.01, with 12.2% reporting low GSE. There is a significant difference between GSE and problem-focused and emotion-focused coping styles (*p* = 0.011; *p* = 0.019, respectively). Unemployment was negatively associated with problem-focused coping (*p* = 0.012), while rural residence and divorce were negatively associated with emotion-focused coping (*p* = 0.037; *p* = 0.004, respectively). Avoidant coping style was determined by unemployment and multiparity (*p* =0.043; *p* = 0.049, respectively). **Conclusions**: These findings highlighted the need for comprehensive perinatal care strategies that incorporate psychosocial factors and address contextual vulnerabilities, particularly among rural, unemployed, and divorced pregnant women.

## 1. Introduction

Pregnancy is a significant life event that is usually accompanied by substantial physical, emotional, and social challenges [1]. It can cause psychological discomfort and expose women to new health risks, especially when support systems are lacking [2,3]. Research showed that pregnant women frequently face complex physiological changes, fluctuating hormones, and greater susceptibility to stress and anxiety [4,5]. In this context, coping mechanisms are key elements in dealing with pregnancy challenges [6]. Coping includes cognitive and behavioral stress management strategies, as well as an appraisal of controllability and personal skills [7]. Coping styles can be functional or dysfunctional depending on personality, culture, and the social context [8].

According to the Social Cognitive Theory, general self-efficacy (GSE) and coping are interconnected [9]. GSE refers to confidence in one’s ability to deal with life’s obstacles [10]. Bandura believes that high GSE enables individuals to activate positive coping, such as problem-solving strategies [9]. Indeed, previous research indicated that GSE correlated positively with effective mechanisms such as positive coping, issue solving, help seeking, and face-to-face coping. In contrast, low GSE showed a negative correlation with ineffective strategies like negative coping, fantasy, self-blaming, avoidance, and yielding [11,12].

In the maternal health context, existing evidence suggests a relationship between self-efficacy and coping during pregnancy [6,13]. For instance, a study among nulliparous pregnant women found that childbirth self-efficacy predicted the use of problem-focused coping styles [14]. However, the available literature remains limited regarding the combined influence of GSE, social determinants, and pregnancy-related factors on coping styles among pregnant women, particularly in lower- and middle-income countries, like Tunisia. Pregnancy in Tunisia is shaped by a combination of maternal health, psychosocial, and sociocultural factors. The country demonstrates relatively strong maternal health indicators, with a maternal mortality ratio of approximately 36 to 37 deaths per 100,000 live births, and nearly 99% of births attended by skilled health personnel [15,16]. Nevertheless, regional inequalities persist in rural and interior areas, where women are facing higher risks due to limited access to specialized services and delays in receiving appropriate care [17]. Psychosocial and sociocultural factors also play a critical role in pregnancy among this population. Recent research found a notable prevalence of low self-efficacy among pregnant women, especially among multiparous women. Key determinants included rural residence, family material well-being, parenting context and overall family quality of life [18].

These facts highlight the need for further research to better understand how these factors interact to shape coping responses during pregnancy and to inform contextually appropriate maternal health strategies. Examining these psychological factors within a theoretical framework is essential for informing perinatal care practices aimed at maintaining emotional well-being during pregnancy and promoting healthier maternal and fetal outcomes. This study aims to identify coping styles among pregnant women attending antenatal care and to examine their relationship with GSE, social characteristics, and pregnancy-related factors.

## 2. Materials and Methods

### 2.1. Settings

This multicenter cross-sectional study was conducted from July 2024 to March 2025 in nine antenatal care clinics in Sousse, a city located in the eastern center of Tunisia (MENA region). The clinics are public sector facilities that provide antenatal care. In Tunisia, the public health sector contains three levels of care: primary care centers and district centers, regional hospitals, and university hospitals. In this study, all levels are targeted at clinics in the center-Est (Sousse region) that provide care for a wide range of populations from all over the country [19].

### 2.2. Participants

The target population includes pregnant women between the ages of 18 and 45 from all socioeconomic groups, regardless of their pregnancy risk status and parity. A multistage sampling approach was employed. In stage 1, researchers randomly selected healthcare centers from a list of 16 centers providing antenatal care in the study region using Excel. The list included different types of facilities, such as a mother and child healthcare center, primary care centers, regional hospitals, and university hospitals, serving a heterogeneous antenatal population. In stage 2, consecutive sampling was employed. Random days were selected for data collection, and on those days, all eligible participants were consecutively recruited, given the relatively low daily number of attendees (5 to 10). Written informed consent was obtained from all participants.

### 2.3. Operational Definition of Variables

#### 2.3.1. Coping Styles

Coping styles are the primary outcomes in this study. They refer to ways in which people typically handle life issues. They are relatively stable traits that guide individuals’ responses to stress [20]. Coping styles are divided into two positive coping styles that include problem-focused coping (efforts to deal directly with the stressor or its source), emotion-focused coping (efforts to reduce emotional reactions to the stressor), and one negative style, which is avoidance coping (efforts to distract from or not think about the stressor) [21].

#### 2.3.2. General Self-Efficacy (GSE)

According to Albert Bandura, self-efficacy is an individual’s belief in their capacity to organize and execute the actions necessary to achieve specific performances or goals. It is measured through domain-specific scales and the General Self-efficacy Scale (GSES) [10].

### 2.4. Data Collection Tools

#### 2.4.1. Sociodemographic and Pregnancy-Related Factors

Participants’ characteristics were assessed using a data collection sheet that included age, area of residence, level of education, employment status, monthly household income, self-reported income level, marital status, gravidity, parity, previous miscarriage, previous cesarean section, planned pregnancy complications, gestational age, and history of chronic diseases. The income level in Tunisia is usually classified using the minimum wage [22], which is defined as approximately 528.320 TND in 2025 (181 USD) [23]. According to the literature, low-income families include workers earning minimum wage or slightly above, middle-income level ranges from 2 to 4 times the minimum wage, offering a moderate standard of living, and high-income level represents more than 4 times the minimum wage [24]. For a broad frame, in this study, we measured the household income level using the minimum wage, as well as the perceived income level.

#### 2.4.2. Brief COPE Inventory

The brief COPE scale is a 28-item questionnaire that measures 14 facets using a 4-point scale [25]. These subscales are grouped into 3 coping styles, including problem-focused coping (Active coping, use of informational support, positive reframing, planning), emotion-focused coping (Emotional Support, venting, humor, Acceptance, self-blame, religion), and avoidant coping (Self-distraction, substance use, denial, behavioral disengagement). Scores are presented for three overarching coping styles as average scores (sum of item scores divided by number of items), indicating the degree to which the respondent has been engaging in that coping style (1 = I haven’t been doing this at all; 2 = A little bit; 3 = A medium amount; 4 = I’ve been doing this a lot). In this study, the Arabic version, trans-culturally validated in the Arabic population with a coefficient of 0.80, was used [26,27]. Studies report acceptable Cronbach’s alpha values, ranging from 0.74 to 0.88 [27,28]. In this study, the overall scale demonstrated good internal consistency, with Cronbach’s alpha of 0.88.

#### 2.4.3. General Self-Efficacy Scale

GSE was measured using the Arabic version of the GSES, which has been validated among Arab women [29]. The GSES is composed of ten items scored on a four-point Likert scale (from 1, “Not at all true,” to 4, “Exactly true”). The total GSE score is calculated by summing the points from each item, with a maximum total score of 40. A higher score indicates higher GSE. It was classified according to the total score as follows: ≤20 indicates low GSE, 21 to 30 indicates moderate GSE, 31 to 40 indicates high GSE. Previous studies have confirmed the high reliability, stability, and construct validity of the GSES among pregnant women. The GSES’s reliability, as measured by Cronbach’s alpha, is 0.85, with internal consistency ranging from 0.76 to 0.91 among pregnant women [30,31]. In this study, Cronbach’s alpha for the GSES was 0.87, indicating good reliability.

### 2.5. Bias

Despite the use of a multi-stage random sampling method to minimize selection bias and enhance the representativeness of the sample, some degree of selection bias may persist due to non-response or exclusion criteria [32]. In addition, validated and reputable instruments with good internal consistency were used to reduce information bias and ensure stable and reproducible measurements. However, reporting bias cannot be entirely ruled out, particularly as some variables rely on self-reported data and may be subject to misreporting or social desirability effects [33]. Furthermore, as with many observational studies, residual confounding remains a concern. Despite adjusting for known confounders, unmeasured or imperfectly measured variables may still influence the observed associations. Finally, the cross-sectional design introduces limitations such as recall bias and averts causal inference.

### 2.6. Study Sample Size

The sample size was calculated using the single population proportion formula. Since the primary outcome, which is coping styles among pregnant women, was unknown and no prior research had been conducted in countries with similar population characteristics, this study used an estimate of 50%, with a 5% margin of error and a 95% confidence level. The minimal sample size was 385. The sample size calculation was based on a single population proportion using a 50% estimate to ensure adequate precision for prevalence estimation. As such, it was not based on outcome-specific effect size assumptions for multivariable linear regression models. This method is intended to provide a sufficiently large and conservative sample to support the overall study objectives.

### 2.7. Statistical Methods

Data entry, descriptive analysis, and score calculation were performed by IBM SPSS Statistics (version 20). The R statistics software (version 4.3.1) was used to perform multivariable linear regression analyses. Quantitative variables were expressed as mean and standard deviation (SD). Qualitative variables were expressed in frequencies and percentages. The normality of continuous variables was assessed using the Kolmogorov–Smirnov test.

Multivariable linear regression was performed to identify predictors of coping styles. Variables were selected for inclusion in the regression models based on theoretical considerations and prior literature rather than univariable statistical screening. All predictors were included in the models, with appropriate adjustment for confounders.

Model assumptions were assessed by examining residual distributions, which indicated no major deviations from normality. Multicollinearity was evaluated using variance inflation factors, with all values within acceptable limits (between 1.02 and 1.22). This fact supports the validity of the models.

Two categories of regression models were performed: primary and exploratory. The primary analyses were defined in advance based on the study objectives and were limited in number. They included three outcomes of coping styles, including avoidant, emotion-focused, and problem-focused coping. As these models represent the main confirmatory analyses of interest, no formal adjustment for multiple comparisons was applied to them.

The exploratory analyses were presented to generate hypotheses for further investigations of potential associations (Supplementary Materials). These models included coping facets as outcomes. Their findings were interpreted cautiously, with an emphasis placed on the magnitude and direction of effect sizes and the consistency of findings across models, rather than statistical significance.

Variables exhibiting sparse categories or evidence of quasi-complete separation were excluded from the multivariable models to avoid unstable parameter estimates. Accordingly, the variable “substance use” was excluded due to minimal variation.

The significance level was defined as *p* < 0.05 with a confidence interval of 95%.

## 3. Results

### 3.1. Information About Sociodemographic and Pregnancy-Related Factors

In total, 417 pregnant women completed the questionnaire. Table 1 shows that the mean age of participants was 30.24 ± 5.41 years, with an average gestational age of 5.88 ± 2.19 months. One hundred thirty-eight women were primigravida (33.1%), one hundred fifty-five (37.2%) were nulliparous, and one hundred thirty-three participants had a previous miscarriage or abortion (31.9%).

### 3.2. Coping Styles and General Self-Efficacy

Results in Table 2 showed that the emotion-focused coping style was mostly reported, with a mean of 31.83 ± 6.85, followed respectively by the problem-focused coping style (22.76 ± 5.65), and the avoidance coping style (14.77 ± 3.70).

In the emotion-focused coping style, religion (6.21 ± 1.73) and emotional support (6.11 ± 1.73) coping facets were mostly adopted by participants. Problem-focused coping included active coping (5.42 ± 1.99), use of informational support (5.85 ± 1.79), positive reframing (5.77 ± 1.80), and planning (5.71 ± 1.71). In avoidant coping, self-distraction (5.06 ± 1.79) was reported by pregnant women in this study.

The GSES reported a mean value of GSE equal to 28.15 ± 6.01. In this study, low GSE was 12.2%.

### 3.3. Factors Related to Coping Styles Among Pregnant Women

Table 3 presents primary analyses of multivariable linear regression. General self-efficacy was positively and significantly associated with problem-focused coping [Estimate: 0.06; 95%CI (0.01, 0.11); *p =* 0.011] and emotion-focused coping [Estimate: 0.07; 95%CI (0.01, 0.13); *p =* 0.019]. Results showed that unemployment was negatively associated with problem-focused coping style [Estimate: −1.46; 95%CI (−2.58, −0.33); *p =* 0.012]. Divorce and rural areas of residence were negatively related to emotion-focused coping, respectively [Estimate: −4.87; 95% CI (−8.17, −1.57); *p =* 0.004], [Estimate: −1.91; 95%CI (−3.69, −0.12); *p =* 0.037].

Exploratory analyses showed that GSE was positively associated with positive reframing, planning, acceptance, and religion coping facets, with a modest effect size and consistency across models (Supplementary Table S3). Regarding social factors, there was a negative association between unemployment and active coping, venting, self-distraction, and planning, with modest-sized effects (Supplementary Tables S1–S3). Rural areas of residence were negatively associated with acceptance and emotional support coping facets. University educational level was positively related to positive reframing with a limited effect size. These exploratory findings were not entirely consistent across models and served to generate hypotheses.

## 4. Discussion

Coping styles play a significant role in maternal psychological well-being and can influence pregnancy outcomes. The insights from this study aim to inform the development of a perinatal care model sensitive to psychosocial and structural influences. Findings indicated that most participants frequently adopt emotion-focused and problem-focused coping styles. The current sample adopted the avoidant coping style less. GSE was positively associated with problem-focused and emotion-focused coping styles. This means that pregnant women with high GSE were more likely to adopt positive coping styles such as problem-focused coping, and vice versa. Similarly, a study conducted among nulliparous pregnant women reported a significant direct correlation between problem-focused coping style and childbirth self-efficacy [14]. Studies among non-pregnant populations, such as university students and patients with chronic illnesses, revealed considerable findings that need to be mentioned. For instance, research conducted among university students suggested that higher levels of self-efficacy were positively associated with problem-focused coping [34]. Studies conducted among chronic patients demonstrated that subjects with high self-efficacy were more likely to use positive coping techniques when dealing with diseases [35,36].

Although the association between GSE and adaptive coping styles was statistically significant in this study, the effect sizes were modest. Nevertheless, these associations remain clinically relevant as even small increases in self-efficacy can enhance coping with stress and adopt healthy behaviors [9]. Such incremental improvements can contribute to better maternal well-being and support the integration of self-efficacy-focused interventions into antenatal care.

Furthermore, the current results showed that divorce and rural areas significantly and negatively correlate with emotion-focused coping. This means that pregnant women living in rural areas and divorced pregnant women may experience greater difficulty in adopting emotional coping styles. This could be explained by the fact that divorce may be accompanied by heightened emotions, feelings of shame, and reduced social support, which may lead to the adoption of emotion-focused coping styles. It is important to note that divorced women may develop greater emotional self-reliance or utilize coping responses not fully captured by emotion-focused coping [37]. Moreover, rural areas in lower-middle-income countries are usually characterized by limited access to antenatal care and psychosocial support services [38], which can explain the negative association between rural areas and emotion-focused coping.

Unemployment significantly and negatively correlates with problem-focused and avoidant coping styles. This data suggested that unemployed women had difficulty coping during pregnancy using problem-focused strategies. From a Social Cognitive Theory perspective, unemployment may reduce perceived self-efficacy and lower perceived control, which can constrain the ability to mobilize effective coping mechanisms [9,10]. Conversely, social factors, such as household income and education, were not associated with coping styles. This finding may be explained by the specific Tunisian context. Indeed, Tunisia has relatively high levels of female education and broad access to primary healthcare services [16,39], which may reduce disparities typically observed in low-resource settings. As a result, basic maternal healthcare, health information, and antenatal services are widely available across different socioeconomic groups. This could potentially minimize the influence of income and education on coping styles.

Avoidant coping was also negatively predicted by multiparity. This suggests that women who have experienced multiple pregnancies are less likely to rely on avoidant coping styles. This finding may indicate that prior pregnancy experience enhances coping resources, reducing reliance on avoidant strategies. The limited literature on the relation of social factors with coping styles during pregnancy restricted the discussion of the current findings.

The current findings suggested that health-related factors, such as maternal age, pregnancy complications, gestational age, previous miscarriage, previous c-section, chronic disease status, and planned pregnancy, were not associated with coping styles. This may be explained by the robustness of antenatal and maternal health services in Tunisia, evidenced by a relatively low maternal mortality ratio, nearly universal skilled birth attendance (approximately 99%), and a substantial proportion of women of reproductive age (62.7%) utilizing modern family planning methods [16].

In this study, the observed associations in primary analyses do not imply causality. Other unmeasured factors, such as social support, mental health status, and cultural influences, may also contribute to coping patterns. The exploratory analyses suggested associations between coping facets, GSE, and various social and pregnancy-related factors. These supplemental findings are not confirmatory and serve as hypotheses generating.

### 4.1. Implications for Practice

These findings suggest the potential need for a holistic perinatal care approach that considers psychosocial mechanisms in antenatal care, in particular GSE and coping. Vulnerable groups, such as rural residents, unemployed and divorced pregnant women, require special attention from primary care providers. Potential implications may include telehealth solutions, mobile clinics, and community outreach to overcome geographic and resource barriers in rural areas. Furthermore, routine psychosocial assessments during antenatal visits may be considered as a potential approach for identifying women with limited emotional support, particularly among divorced pregnant women. Counseling services and peer support groups may offer opportunities to support self-efficacy and coping among pregnant women.

### 4.2. Limitations

Several limitations should be considered when interpreting the findings. First, the cross-sectional design limits causal inference and introduces recall bias. Second, despite using a random sampling from multiple settings, the study is considered region-specific, which limits statistical inference. Moreover, selection bias, reporting bias and residual confounding cannot be entirely excluded. Although validated instruments were employed, the possibility of shared method variance cannot be excluded. Consequently, some observed associations may have been inflated due to the use of a common measurement method rather than solely reflecting the true relationships between the studied constructs. Data analyses were structured into primary and exploratory components to address concerns about multiple testing. However, multiple exploration models may still increase the risk of a Type I error. Therefore, statistically significant findings from exploration analyses were interpreted cautiously and not considered confirmatory. These limitations highlight the importance of cautious interpretation and confirmation of the current findings in future studies.

## 5. Conclusions

The current study identified key patterns of coping and associations with GSE and social factors among pregnant women in a lower-middle-income country. The findings provided insights that can inform perinatal care practice, shape preventive programs, and contribute to improving overall maternal mental health during pregnancy. Targeted strategies that address GSE, particularly among rural residents, divorced and unemployed pregnant women, may activate positive coping styles among this population.

## Figures and Tables

**Table 1:** Sociodemographic and clinical information of pregnant women (n = 417).

Variables		n (%)
Area of residency	Urban Rural	333 (79.9) 84 (20.1)
Level of education	Illiterate Primary Secondary University	19 (4.6) 55 (13.2) 164 (39.3) 179 (42.9)
Employment status	Employed Unemployed	231 (55.4) 186 (44.6)
Household monthly income	MW * or slightly above Two to four MW More than 4 MW	185 (44.4) 176 (42.2) 56 (13.4)
Household perceived income level	Low Moderate High	75 (18) 319 (76.5) 23 (5.5)
Marital status	Married Divorced Widowed	397 (95.2) 18 (4.3) 2 (.5)
Gravida	Primigravida Multigravida	138 (33.1) 279 (66.9)
Parity	Nulliparous Multiparous	155 (37.2) 262 (62.8)
Having chronic diseases	No Yes	332 (79.6) 85 (20.4)
Previous miscarriage	No Yes	284 (68.1) 133 (31.9)
Previous C-section	No Yes	289 (69.3) 128 (30.7)
Planned pregnancy	No Yes	173 (41.5) 244 (58.5)
Pregnancy complications	No Yes	269 (64.5) 148 (35.5)
Gestational age in months (m ± SD)		5.88 ± 2.19
Women age in years (m ± SD)		30.24 ± 5.41

* MW: Minimum wage.

**Table 2:** Results from the COPE-Brief Inventory and the General Self-efficacy Scale among 417 pregnant women in Tunisia.

Scales and Subscales	m ± SD
Problem-Focused Coping Style	22.76 ± 5.65
Active coping	5.42 ± 1.99
Use of informational support	5.85 ± 1.79
Positive reframing	5.77 ± 1.80
Planning	5.71 ± 1.71
Emotion-Focused Coping Style	31.83 ± 6.85
Emotional support	6.11 ± 1.73
Venting	5.13 ± 1.85
Humor	4.21 ± 1.80
Acceptance	5.99 ± 1.58
Religion	6.21 ± 1.73
Self-blame	4.15 ± 1.84
Avoidant Coping Style	14.77 ± 3.70
Self-distraction	5.06 ± 1.79
Denial	3.51 ± 1.63
Substance use	2.29 ± 0.91
Behavioral disengagement	3.88 ± 1.63
General Self-efficacy Scale	28.15 ± 6.016
GSE levels, n (%)	
Low	52 (12.4)
Moderate	220 (52.8)
High	145 (34.8)

**Table 3:** Primary analyses: Multivariable linear regression models assessing the association between coping styles and general self-efficacy, sociodemographic and pregnancy-related factors among pregnant women attending antenatal care in Tunisia (n = 417).

	Problem-Focused Coping Style *			Emotion-Focused Coping Style *			Avoidant Coping Style *		
Covariates	Estimate	95% CI	*p*-Value	Estimate	95% CI	*p*-Value	Estimate	95% CI	*p*-Value
Age	0.02	(−0.09, 0.13)	0.762	−0.08	(−0.21, 0.06)	0.281	−0.06	(−0.13, 0.02)	0.151
Educational level									
No education	Reference				Reference		Reference		
Primary	2.52	(−0.44, 5.47)	0.096	1.99	(−1.65, 5.59)	0.287	−0.03	(−2.06, 2.00)	0.978
Secondary	2.25	(−0.47, 4.89)	0.106	1.26	(−2.09, 4.60)	0.462	−0.31	(−2.19, 1.57)	0.745
Tertiary	2.28	(−0.47, 5.04)	0.105	1.83	(−1.55, 5.20)	0.288	−0.13	(−2.02, 1.76)	0.891
Marital status									
Married	Reference				Reference		Reference		
Divorced	−2.65	(−5.34, 0.04)	0.055	−4.87	(−8.17, −1.57)	0.004	−1.59	(−3.39, 0.21)	0.891
Widowed	−0.62	(−8.31, 7.08)	0.875	0.91	(−8.52, 10.35)	0.849	0.96	(−4.19, 6.13)	0.713
Employment status									
Employed	Reference				Reference		Reference		
Unemployed	−1.46	(−2.58, −0.33)	0.012	−1.07	(−2.45, 0.31)	0.130	−0.79	(−1.54, −0.02)	0.043
Place of residence									
Urban	Reference				Reference		Reference		
Rural	−0.88	(−2.34, 0.58)	0.239	−1.91	(−3.69, −0.12)	0.037	−0.38	(−1.35, 0.60)	0.453
Household income level									
Low	Reference				Reference		Reference		
Moderate	−0.33	(−1.81, 1.16)	0.659	−0.63	(−2.44, 1.18)	0.495	−0.89	(−1.88, 0.10)	0.080
High	1.93	(−0.79, 4.65)	0.166	−0.31	(−3.64, 3.03)	0.858	−0.42	(−2.25, 1.40)	0.649
Parity									
Nulliparous	Reference				Reference		Reference		
Multiparous	−1.00	(−2.33, 0.33)	0.139	−0.94	(−2.57, 0.69)	0.260	−0.89	(−1.79, −0.00)	0.049
Having chronic diseases									
No	Reference				Reference		Reference		
Yes	−0.01	(−1.39, 1.37)	0.987	0.12	(−1.50, 1.89)	0.821	0.31	(−0.62, 1.24)	0.517
Gestational age	0.19	(−0.06, 0.43)	0.135	0.17	(−0.13, 0.47)	0.268	0.09	(−0.06, 1.24)	0.250
Complications during pregnancy									
No	Reference				Reference		Reference		
Yes	−0.71	(−1.88, 0.46)	0.234	−0.62	(−2.06, 0.81)	0.397	0.39	(−0.40, 1.17)	0.336
Previous miscarriage									
No	Reference				Reference		Reference		
Yes	0.26	(−0.91, 1.43)	0.658	0.51	(−0.92, 1.95)	0.482	−0.05	(−0.83, 0.74)	0.908
Previous c-section									
No	Reference				Reference		Reference		
Yes	1.31	(−0.02, 2.65)	0.054	1.22	(−0.42, 2.86)	0.145	0.71	(−0.18, 1.61)	0.119
Planned pregnancy									
No	Reference				Reference		Reference		
Yes	0.83	(−0.29, 1.94)	0.148	−0.13	(−1.50, 1.24)	0.852	−0.09	(−0.84, 0.66)	0.813
General self-efficacy	0.06	(0.01, 0.11)	0.011	0.07	(0.01, 0.13)	0.019	0.00	(−0.03, 0.04)	0.809

* Variance Inflation Factors for all models are between 1.02 and 1.22.

## Data Availability

The dataset generated and analyzed during the current study is available in the Mendeley Data repository (Link: https://data.mendeley.com/datasets/zsmb2xsdmx/7; accessed on 24 June 2025).

## Supplementary Materials

The following supporting information can be downloaded at: https://www.mdpi.com/article/10.3390/healthcare14131977/s1. Supplementary Table S1: Multivariable linear regression analyses assessing the association of general self-efficacy and the coping facets of the COPE-Brief questionnaire among pregnant women attending antenatal care (n = 417); Supplementary Table S2: Multivariable linear regression analyses assessing the association of general self-efficacy and the coping facets of the COPE-Brief questionnaire among pregnant women attending antenatal care (n = 417); Supplementary Table S3: Multivariable linear regression analyses assessing the association of coping facets of the COPE-Brief questionnaire with participants’ characteristics and general self-efficacy among pregnant women attending antenatal care in Tunisia (n = 417); Supplementary Table S4: Multivariable linear regression analyses assessing the association of general self-efficacy and the coping facets of the COPE-Brief questionnaire among pregnant women attending antenatal care (n = 417).

## Abbreviations

The following abbreviations are used in this manuscript:

GSE	General self-efficacy
GSES	General self-efficacy scale
