# Supplementary material for: Coping Styles and General Self-Efficacy Among Pregnant Women: Evidence from a Multicenter Study in Tunisia
Source: Healthcare (Basel). 2026 Jul 2;14(13):1977. doi: 10.3390/healthcare14131977 (PMC13362182; doi:10.3390/healthcare14131977)
Supplement: Supplementary file 1 [file healthcare-14-01977-s001.zip › healthcare-4288911-supplementary.pdf]

Supplementary Table S1*:* Multivariable linear regression analyses assessing the association of general self-efficacy and the coping facets of the COPE-Brief questionnaire among pregnant women attending antenatal care (n=417).

	**Multivariable model 1***			**Multivariable model 2****			**Multivariable model 3*****			**Multivariable model 4******		
**Covariates**	**Estimate**	**95% CI**	**p-value**	**Estimate**	**95% CI**	**p-value**	**Estimate**	**95% CI**	**p-value**	**Estimate**	**95% CI**	**p-value**
**Age**	0.00	(-0.03, 0.04)	0.933	0.03	(-0.02, 0.05)	0.133	-0.02	(-0.05, 0.02)	0.328	-0.02	(-0.06, 0.01)	0.205
**Educational level**												
No education		Reference			Reference			Reference			Reference	
Primary	0.49	(-0.44, 1.44)	0.299	1.04	(-0.02, 2.10)	0.055	0.82	(-0.08, 1.72)	0.076	0.10	(-0.88, 1.08)	0.837
Secondary	0.59	(-0.28, 1.45)	0.186	0.29	(-0.67, 1.27)	0.551	0.77	(-0.06, 1.60)	0.071	0.24	(-0.67, 1.14)	0.610
Tertiary	0.54	(-0.33, 1.41)	0.227	0.36	(-0.62, 1.35)	0.470	0.95	(0.11, 1.79)	**0.027**	0.32	(-0.59, 1.23)	0.499
**Marital status**												
Married		Reference			Reference			Reference			Reference	
Divorced	-0.64	(-1.49, 0.22)	0.145	-0.07	(-1.04, 0.89)	0.879	-0.68	(-1.51, -0.14)	0.104	-1.27	(-2.16, -0.38)	**0.005**
Widowed	0.21	(-2.23, 2.65)	0.866	-1.29	(-4.05, 1.46)	0.357	0.87	(-1.48, 3.22)	0.469	0.14	(-2.41, 2.70)	0.913
**Employment status**												
Employed		Reference			Reference			Reference			Reference	
Unemployed	-0.16	(-0.52, 0.19)	0.385	-0.67	(-1.08, 0.27)	**0.001**	-0.04	(-0.39, 0.30)	0.809	-0.43	(-0.81, -0.05)	**0.025**
**Place of residence**												
Urban		Reference			Reference			Reference			Reference	
Rural	-0.44	(-0.91, 0.02)	0.061	-0.13	(-0.65, 0.39)	0.638	-0.48	(-0.92, -0.03)	**0.036**	-0.10	(-0.59, 0.38)	0.673
**Household income level**												
Low		Reference			Reference			Reference			Reference	
Moderate	-0.04	(-0.51, 0.43)	0.863	-0.09	(-0.62, 0.44)	0.741	-0.02	(-0.47, 0.43)	0.929	-0.48	(-0.97, 0.01)	0.054
High	0.37	(-0.49, 1.23)	0.403	0.44	(-0.54, 1.4)	0.381	0.39	(-0.43, 1.22)	0.349	-0.39	(-1.29, 0.51)	0.395
**Parity**												
Nulliparous		Reference			Reference			Reference			Reference	
Multiparous	-0.17	(-0.59, 0.25)	0.429	-0.28	(-0.75, 0.12)	0.254	-0.27	(-0.68, 0.14)	0.193	-0.03	(-0.48, 0.41)	0.877
**Having chronic diseases**												
No		Reference			Reference			Reference			Reference	
Yes	-0.19	(-0.63, 0.24)	0.384	0.03	(-0.46, 0.53)	0.896	-0.21	(-0.63, 0.22)	0.337	-0.09	(-0.55, 0.37)	0.709

**Gestational age**	0.05	(-0.03, 0.12)	0.254	0.01	(-0.07, 0.09)	0.896	0.06	(-0.01, 0.14)	0.109	0.02	(-0.06, 0.10)	0.583
**Complications during pregnancy**												
No		Reference			Reference			Reference			Reference	
Yes	-0.48	(-0.85, -0.12)	**0.012**	0.11	(-0.31, 0.53)	0.614	-0.28	(-0.64, 0.07)	0.119	-0.30	(-0.69, 0.09)	0.131
**Previous miscarriage**												
No		Reference			Reference			Reference			Reference	
Yes	-0.28	(-0.65, 0.09)	0.134	0.16	(-0.26, 0.57)	0.464	-0.24	(-0.59, 0.12)	0.192	0.01	(-0.37, 0.40)	0.943
**Previous c-section**												
No		Reference			Reference			Reference			Reference	
Yes	0.46	(0.04, 0.88)	**0.034**	0.27	(-0.19, 0.76)	0.252	0.38	(-0.03, 0.79)	0.069	0.19	(-0.25, 0.63)	0.401
**Planned pregnancy**												
No		Reference			Reference			Reference			Reference	
Yes	0.35	(-0.00, 0.71)	0.052	-0.11	(-0.51, 0.29)	0.590	0.30	(-0.04, 0.64)	0.083	-0.18	(-0.55, 0.19)	0.338
**General self-efficacy**	0.01	(-0.00, 0.03)	0.176	0.01	(-0.01, 0.03)	0.250	0.01	(-0.00, 0.03)	0.165	0.02	(-0.00, 0.03)	0.076

*Model 1 used the use of informational support as the outcome of interest.

** Model 2 used active coping as the outcome of interest.

*** Model 3 used emotional support as the outcome of interest.

****Model 4 used venting as the outcome of interest.

Supplementary Table S2*:* Multivariable linear regression analyses assessing the association of general self-efficacy and the coping facets of the COPE-Brief questionnaire among pregnant women attending antenatal care (n=417).

	**Multivariable model 5***			**Multivariable model 6****			**Multivariable model 7*****			**Multivariable model 8******		
**Covariates**	**Estimate**	**95% CI**	**p-value**	**Estimate**	**95% CI**	**p-value**	**Estimate**	**95% CI**	**p-value**	**Estimate**	**95% CI**	**p-value**
**Age**	-0.02	(-0.06, 0.01)	0.194	0.02	(-0.02, 0.05)	0.424	-0.02	(-0.05, 0.01)	0.315	-0.02	(-0.06, 0.01)	0.184
**Educational level**												
No education		Reference			Reference			Reference			Reference	
Primary	0.36	(-0.61, 1.32)	0.469	0.08	(-0.91, 1.07)	0.869	0.21	(-0.69, 1.11)	0.651	-0.04	(-0.91, 0.84)	0.935
Secondary	-0.06	(-0.95, 0.82)	0.886	-0.23	(-1.14, 0.69)	0.628	0.54	(-0.29, 1.37)	0.207	-0.23	(-1.04, 0.58)	0.582
Tertiary	0.07	(-0.82, 0.97)	0.872	-0.34	(-1.26, 0.58)	0.469	0.69	(-0.16, 1.52)	0.116	-0.16	(-0.98, 0.65)	0.692
**Marital status**												
Married		Reference			Reference			Reference			Reference	
Divorced	-1.00	(-1.88, -0.12)	**0.026**	-0.70	(-1.61, 0.19)	0.123	-1.02	(-1.84, -0.19)	**0.016**	-0.41	(-1.20, 0.39)	0.319
Widowed	0.29	(-2.22, 2.79)	0.821	0.38	(-2.19, 2.95)	0.773	-0.93	(-3.29, 1.42)	0.438	-0.42	(-2.70, 1.86)	0.716
**Employment status**												
Employed		Reference			Reference			Reference			Reference	
Unemployed	-0.19	(-0.56, 0.17)	0.303	-0.11	(-0.49, 0.27)	0.567	-0.81	(-1.16, 0.47)	**<0.001**	0.09	(-0.25, 0.42)	0.609
**Place of residence**												
Urban		Reference			Reference			Reference			Reference	
Rural	-0.46	(-0.94, 0.01)	0.05	-0.14	(-0.63, 0.34)	0.557	-0.39	(-0.84, 0.05)	0.081	-0.09	(-0.52, 0.34)	0.687
**Household income level**												
Low		Reference			Reference			Reference			Reference	
Moderate	-0.26	(-0.74, 0.22)	0.286	-0.35	(-0.84, 0.14)	0.162	-0.19	(-0.65, 0.25)	0.389	-0.38	(-0.82, 0.05)	0.086
High	-0.48	(-1.37, 0.40)	0.286	-0.96	(-1.86, -0.03)	**0.043**	0.43	(-0.40, 1.26)	0.314	-0.78	(-1.59, 0.02)	0.059
**Parity**												
Nulliparous		Reference			Reference			Reference			Reference	
Multiparous	-0.12	(-0.55, 0.31)	0.579	-0.27	(-0.72, 0.17)	0.235	-0.11	(-0.52, 0.29)	0.600	-0.33	(-0.72, 0.07)	0.105
**Having chronic diseases**												
No		Reference			Reference			Reference			Reference	

Yes	0.47	(0.01, 0.92)	**0.043**	0.07	(-0.39, 0.53)	0.235	0.33	(-0.09, 0.76)	0.122	0.19	(-0.22, 0.60)	0.360
**Gestational age**	-0.01	(-0.09, 0.07)	0.749	0.02	(-0.06, 0.10)	0.619	0.09	(0.02, 0.17)	**0.013**	-0.02	(-0.09, 0.06)	0.658
**Complications during pregnancy**												
No		Reference			Reference			Reference			Reference	
Yes	0.04	(-0.33, 0.42)	0.820	0.19	(-0.21, 0.58)	0.354	-0.23	(-0.59, 0.13)	0.204	0.46	(0.07, 0.77)	**0.017**
**Previous miscarriage**												
No		Reference			Reference			Reference			Reference	
Yes	0.13	(-0.25, 0.51)	0.512	0.22	(-0.17, 0.61)	0.27	0.07	(-0.29, 0.42)	0.698	-0.07	(-0.41, 0.28)	0.710
**Previous c-section**												
No		Reference			Reference			Reference			Reference	
Yes	0.19	(-0.25, 0.62)	0.403	0.05	(-0.39, 0.50)	0.819	-0.11	(-0.52, 0.29)	0.593	0.38	(-0.05, 0.73)	0.095
**Planned pregnancy**												
No		Reference			Reference			Reference			Reference	
Yes	0.07	(-0.29, 0.43)	0.714	-0.37	(-0.74, 0.01)	0.055	0.16	(-0.19, 0.49)	0.375	-0.09	(-0.42, 0.24)	0.592
**General self-efficacy**	0.01	(0.00, 0.03)	0.059	-0.00	(-0.02, 0.01)	0.432	0.01	(-0.00, 0.03)	0.074	-0.00	(-0.02, 0.01)	0.658

*Model 5 used humor as the outcome of interest.

** Model 6 used self-blame as the outcome of interest.

*** Model 7 used self-distraction as the outcome of interest.

****Model 8 used denial as the outcome of interest.

Supplementary Table S3*:* Multivariable linear regression analyses assessing the association of coping facets of the COPE-Brief questionnaire with participants' characteristics and general self-efficacy among pregnant women attending antenatal care in Tunisia (n=417).

	Multivariable model 9*			Multivariable model 10**			Multivariable model 11***			Multivariable model 12 ****		
**Covariates**	**Estimate**	**95% CI**	**p-value**	**Estimate**	**95% CI**	**p-value**	**Estimate**	**95% CI**	**p-value**	**Estimate**	**95% CI**	**p-value**
**Age**	-0.03	(-0.06, 0.01)	0.117	0.01	(-0.02, 0.05)	0.419	-0.01	(-0.04, 0.02)	0.496	-0.01	(-0.05, 0.02)	0.413
**Educational level**												
**No education**		Reference			Reference			Reference			Reference	
**Primary**	0.61	(-0.34, 1.56)	0.210	0.37	(-0.51, 1.25)	0.407	0.52	(-0.30, 1.34)	0.216	0.09	(-0.85, 1.02)	0.856
**Secondary**	0.86	(-0.02, 1.73)	0.056	0.51	(-0.30, 1.32)	0.217	0.41	(-.035, 1.71)	0.292	0.13	(-0.73, 0.99)	0.767
**Tertiary**	0.93	(0.04, 1.81)	**0.040**	0.42	(-0.37, 1.27)	0.283	0.51	(-0.26, 1.27)	0.197	0.32	(-0.55, 1.19)	0.471
**Marital status**												
**Married**		Reference			Reference			Reference			Reference	
**Divorced**	-0.73	(-1.59, 0.14)	0.099	-1.21	(-2.01, -0.41)	**0.003**	-0.94	(-1.69, -0.19)	**0.015**	-0.27	(-1.12, 0.58)	0.537
**Widowed**	0.94	(-1.53, 3.41)	0.456	-0.47	(-2.77, 1.82)	0.688	-0.85	(-2.99, 1.29)	0.438	0.09	(-2.35, 2.52)	0.945
**Employment status**												
**Employed**		Reference			Reference			Reference			Reference	
**Unemployed**	-0.18	(-0.55, 0.18)	0.324	-0.44	(-0.78, -0.10)	**0.011**	-0.18	(-0.49, 0.14)	0.266	-0.11	(-0.47, 0.24)	0.529
**Place of residence**												
**Urban**		Reference			Reference			Reference			Reference	
**Rural**	-0.07	(-0.54, 0.39)	0.770	-0.24	(-0.67, 0.19)	0.283	-0.49	(-0.89, -0.81)	**0.019**	-0.23	(-0.69, 0.24)	0.337
**Household income level**												
**Low**		Reference			Reference			Reference			Reference	
**Moderate**	-0.06	(-0.54, 0.43)	0.796	-0.14	(-0.58, 0.30)	0.536	0.29	(-0.11, 0.71)	0.156	0.19	(-0.28, 0.66)	0.431
**High**	0.62	(-0.25, 1.49)	0.165	0.50	(-0.31, 1.31)	0.225	0.69	(-0.07, 1.45)	0.075	0.42	(-0.43, 1.29)	0.334
**Parity**												
**Nulliparous**		Reference			Reference			Reference			Reference	
**Multiparous**	-0.23	(-0.65, 0.20)	0.299	-0.33	(-0.73, 0.07)	0.103	-0.21	(-0.58, 0.17)	0.277	-0.03	(-0.45, 0.38)	0.874
**Having chronic diseases**												
**No**		Reference			Reference			Reference			Reference	

**Yes**	0.30	(-0.14, 0.75)	0.184	-0.15	(-0.56, 0.26)	0.473	-0.07	(-0.46, 0.32)	0.726	0.02	(-0.41, 0.46)	0.914
**Gestational age**	0.07	(-0.01, 0.15)	0.079	0.06	(-0.01, 0.13)	0.112	0.06	(-0.01, 0.12)	0.116	0.02	(-0.05, 0.10)	0.558
**Complications during pregnancy**												
**No**		Reference			Reference			Reference			Reference	
**Yes**	-0.15	(-0.53, 0.22)	0.422	-0.19	(-0.54, 0.16)	0.293	-0.16	(-0.48, 0.17)	0.351	-0.11	(-0.48, 0.26)	0.561
**Previous miscarriage**												
**No**		Reference			Reference			Reference			Reference	
**Yes**	0.18	(-0.19, 0.55)	0.352	0.21	(-0.14, 0.56)	0.231	0.19	(-0.13, 0.53)	0.230	0.19	(-0.18, 0.56)	0.313
**Previous c-section**												
**No**		Reference			Reference			Reference			Reference	
**Yes**	0.14	(-0.29, 0.57)	0.521	0.43	(0.03, 0.83)	0.035	0.33	(-0.04, 0.71)	0.081	0.08	(-0.35, 0.50)	0.716
**Planned pregnancy**												
**No**		Reference			Reference			Reference			Reference	
**Yes**	0.18	(-0.18, 0.54)	0.329	0.40	(0.07, 0.74)	0.018	0.14	(-0.17, 0.45)	0.384	-0.09	(-0.44, 0.26)	0.619
**General self-efficacy**	0.019	(0.004, 0.03)	**0.016**	0.02	(0.01, 0.04)	**0.002**	0.02	(0.01, 0.03)	**0.005**	0.02	(0.003, 0.03)	**0.023**

*Model 9 used positive reframing as the outcome of interest.

** Model 10 used planning as the outcome of interest.

*** Model 11 used acceptance as the outcome of interest.

****Model 12 used religion as the outcome of interest.

Supplementary Table S4: Multivariable linear regression analyses assessing the association of general self-efficacy and the coping facets of the COPE-Brief questionnaire among pregnant women attending antenatal care (n=417).

	Multivariable model 13*		
**Covariates**	**Estimate**	**95% CI**	**p-value**
**Age**	-0.01	(-0.05, 0.02)	0.902
**Educational level**			
No education	Reference		
Primary	0.27	(-0.62, 1.17)	0.549
Secondary	-0.19	(-1.02, 0.64)	0.655
Tertiary	-0.28	(-1.12, 0.55)	0.509
**Marital status**			
Married	Reference		
Divorced	-0.19	(-0.99 0.60)	0.633
Widowed	-0.33	(-2.61, 1.95)	0.77
**Employment status**			
Employed	Reference		
Unemployed	-0.04	(-0.37, 0.29)	0.816
**Place of residence**			
Urban	Reference		
Rural	0.01	(-0.42, 0.45)	0.953
**Household income level**			
Low	Reference		
Moderate	-0.44	(-0.88, 0.00)	0.052
High	0.12	(-0.68, 0.93)	0.765
**Parity**			
Nulliparous	Reference		
Multiparous	-0.35	(-0.74, 0.05)	0.085
**Having chronic diseases**			
No	Reference		
Yes	-0.19	(-0.59, 0.22)	0.372
**Gestational age**	0.06	(-0.01, 0.14)	0.078
**Complications during pregnancy**			
No	Reference		

Yes	0.20	(-0.15, 0.58)	0.256
**Previous miscarriage**			
No	Reference		
Yes	0.09	(-0.27, 0.42)	0.658
**Previous c-section**			
No	Reference		
Yes	0.37	(-0.02, 0.77)	0.066
**Planned pregnancy**			
No	Reference		
Yes	-0.12	(-0.45, 0.22)	0.496
**General self-efficacy**	-0.01	(0.02, 0.01)	0.468
* Model 13 used behavioral disengagement the outcome of interest.			
